# The first 3D analysis of the sphenoid morphogenesis during the human embryonic period

**DOI:** 10.1038/s41598-022-08972-w

**Published:** 2022-03-28

**Authors:** Natsuko Utsunomiya, Motoki Katsube, Yutaka Yamaguchi, Akio Yoneyama, Naoki Morimoto, Shigehito Yamada

**Affiliations:** 1 Department of Plastic and Reconstructive Surgery, Graduate School of Medicine, Kyoto University, 54 Kawahara-cho, Shogoin, Sakyo-ku, Kyoto, 606-8507 Japan; 2 Congenital Anomaly Research Center, Graduate School of Medicine, Kyoto University, Kyoto, Japan; 3 SAGA Light Source, Saga, Japan; 4 Human Health Sciences, Graduate School of Medicine, Kyoto University, Kyoto, Japan

**Keywords:** Embryology, Morphogenesis, Bone

## Abstract

The sphenoid has a complicated shape, and its morphogenesis during early development remains unknown. We aimed to elucidate the detailed morphogenesis of the sphenoid and to visualize it three-dimensionally using histological section (HS) and phase-contrast X-ray CT (PCX-CT). We examined 65 specimens using HS and 57 specimens using PCX-CT, and summarized the initial morphogenesis of the sphenoid during Carnegie stage (CS) 17 to 23. The 3D models reconstructed using PCX-CT demonstrated that some neural foramina have the common process of "neuro-advanced" formation and revealed that shape change in the anterior sphenoid lasts longer than that of the posterior sphenoid, implying that the anterior sphenoid may have plasticity to produce morphological variations in the human face. Moreover, we measured the cranial base angle (CBA) in an accurate midsagittal section acquired using PCX-CT and found that the CBA against CS was largest at CS21. Meanwhile, CBA against body length showed no striking peak, suggesting that the angulation during the embryonic period may be related to any developmental events along the progress of stages rather than to a simple body enlargement. Our study elucidated the normal growth of the embryonic sphenoid, which has implications for the development and evolution of the human cranium.

## Introduction

### The sphenoid and human craniofacial development

The human skull is unique amongst mammals in that it has a large and spherical calvarium and an upright and retruded face^1^. The cranial base, which anatomically lies just between the brain and the face, can be intensely studied as the origin of such human-like skull features^2,3^. A noticeable morphological feature of the human cranial base is its acute angle bent toward the cranial vault side, which is one of the critical findings when studying the evolution of the human cranium, involving brain enlargement^2–5^, the pharyngeal and paranasal cavity^2,6^, or human-specific posture or locomotion^7^.

The sphenoid is one of the principal components of the cranial base with a very distinctive shape. Scott emphasized the importance of the sphenoid by referring to it as the primary region responsible for cranial base shape variation^8^. There are several important synchondroses around the sphenoid, and it is suggested that the different timing of ossification of each synchondrosis may result in the degree of cranial base angulation^2,8,9^. Lieberman proposed that the sphenoid shortening in the sagittal midline in humans could alter the spatial relationship between the cranial base, face, and cranial vault, and moreover determine the protrusion of the face^10^. In contrast, the geometric analysis by Bastir & Rosas^11^ indicated that the relative position between the sphenoidal wings and the midline of the cranial base, rather than the length of the sphenoid, may contribute to the projection of the maxilla and zygoma. Thus, it can be inferred that the sphenoid is a controversial and significant structure regarding not only the morphogenesis of the cranial base but also the features of a human-specific cranium and face. Furthermore, it has been reported that malformations of the sphenoidal region can be observed in many congenital craniofacial deformities, including craniosynostosis, cleft lip and palate^12,13^, Down syndrome^14–16^, and others^17,18^, which emphasize the importance of the sphenoid in the pathogenesis of these anomalies. Recent advances in imaging techniques have led to the realization of the significance of the sphenoid, including evolutionary and developmental aspects, and many anatomists have been keenly interested in the unique shape of the sphenoid as it has been called by various names since ancient Greece^19^; however, the detailed process of the morphogenesis of the sphenoid itself remains unknown.

### Previous research on the morphogenesis of the sphenoid

The development of the chondrocranium, including the sphenoid, consists of the following phases: mesenchymal cell migration and proliferation, epithelial interaction, mesenchymal condensation, production of a cartilaginous matrix and perichondrium (chondrification), and ossification^20–22^. The sphenoid originates from the mesoderm and neural crest cells during the fourth week of development^20^. Mesenchymal cells proliferate to form three precursors, namely, the lateral cartilage, hypophyseal cartilage, and prechordal cartilage^23–25^. These differentiate into the primary cartilaginous elements of the sphenoid at the end of the embryonic period, which play the role of a scaffold for subsequent cranial formation^22,26^. After 8 or 9-weeks of gestational age (the fetal period), cartilaginous elements are subsequently replaced with bony structures in two different ways: intermembranous or endochondral ossification. The process of ossification is now well known based on previous studies on human fetuses using computed tomography (CT)^26^ or magnetic resonance imaging (MRI)^27^. On the other hand, how the primary elements of the sphenoid arise and develop during 6 to 8-weeks of gestational age has not been fully understood due to the lack of sufficient samples and observation devices. For instance, in 1910, Fawcett ^28^ performed a detailed histological observation using 19 mm and 30 mm crown-rump length (CRL) human embryos, and sketched the sphenoid of these specimens. Kernan^29^ studied the chondrocranium of a 20 mm human embryo, further describing the anatomy of the primordial sphenoid, including its positional relationship with other organs. Müller & O’rahilly^30^ added a histological study of the chondrocranium of human embryos ranging from 27 to 32 mm in CRL, with special regard to the relationship with the nervous system. Yamamoto^31^ performed a histological study focused on the connection between the alar process and the ala temporalis, and reconstructed 3D models of a part of the sphenoid. These studies were time-discrete or location-limited, and most of them neither elucidated the continuous growth nor the three-dimensional (3D) morphogenesis of the initial development of the sphenoid.

### Imaging devices for human embryos

Most morphological analyses of human embryos have been performed in 2D in previous studies, which have made a substantial contribution to establish the foundation of embryology. For comparison with previous 2D analysis, the benefits of studying 3D volume data are as follows: (1) 3D visualization, (2) observation of a complicated structure from arbitrary angles, and (3) obtaining an accurate anatomical plane (e.g., sagittal plane as in this study) which allows for morphometric analysis with higher accuracy. Furthermore, from the perspective of preserving invaluable specimens, morphometric studies on embryos and fetuses are shifting from using anatomical experiments or tissue sections to non-destructive imaging methods.

The 3D imaging techniques which can be applied to embryos are CT and MRI. MRI is a favorable device for human fetal samples, as used in previous studies^32–35^. However, a spatial resolution of 40 µm is provided by 7 Tesla MRI, which is not minute enough to observe individual embryonic organs. Conventional CT has a relatively good resolution, but it uses the X-ray absorption of a sample. We are not able to investigate the primordial skull using conventional CT because most components in embryos remain as cartilages with low X-ray absorption^27^. Phase-contrast CT (PCX-CT) captures the X-ray “phase-shift”, which is generated when the X-ray passes through a sample^32,36^. The sensitivity of PCX-CT for light elements, such as carbon, nitrogen, and oxygen is approximately 1000 times higher than that of conventional X-ray CT^36^. As these features are suitable for soft materials such as embryos, PCX-CT makes it possible to investigate the embryonic sphenoid three dimensionally, which cannot be observed equally well using conventional X-ray CT.

### Purpose of this study

The primary purpose of this study was to observe the morphology of the sphenoid during the embryonic period using histological section (HS) and PCX-CT images and to establish the normal process of the early development of the sphenoid. Second, we aimed to visualize the embryonic sphenoid under development by reconstructing 3D models using PCX-CT and to reveal its continuous morphological changes. We expect that a detailed description of the morphogenesis of the sphenoid may give us insights into the development of the human skull and face, as well as the evolution of the human cranium and the pathogenesis of congenital craniofacial diseases.

## Results

**Figure 1:**
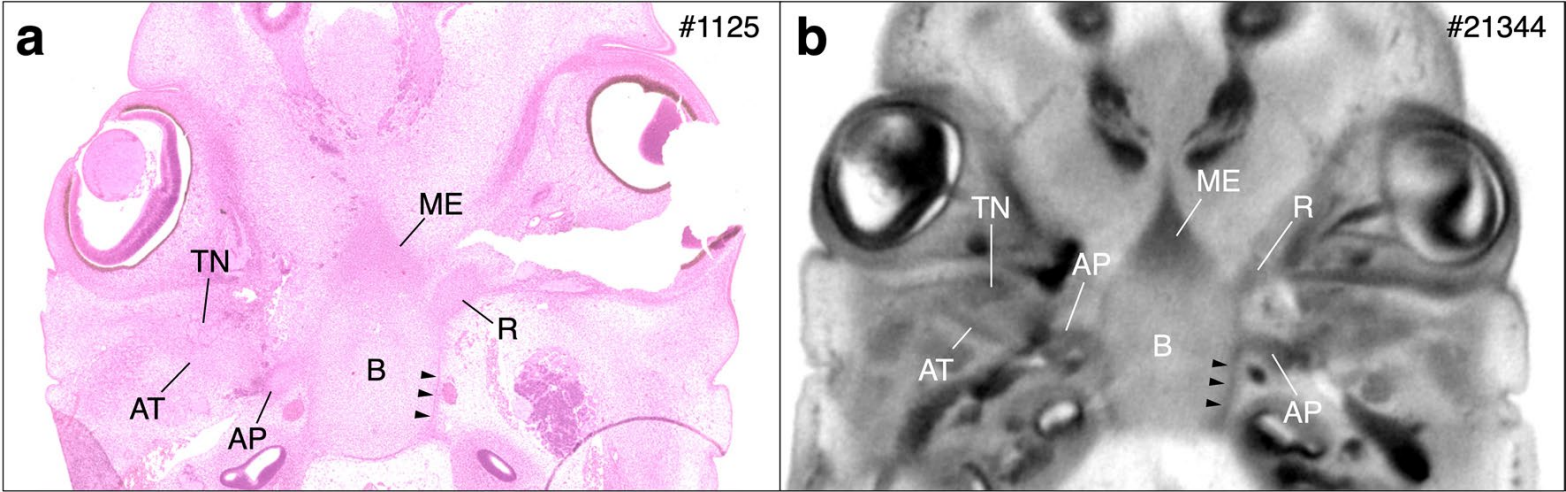
An example of cartilaginous elements in CS20 are observed using HS (**a**) and PCX-CT (**b**). These specimens include both cartilaginous matrix and condensation. Elements with chondrified matrix surrounded by perichondrium (e.g., basisphenoid) in HS are observed as low-density areas surrounded by a high-density outline in PCX-CT. Arrow heads show the perichondrium. Condensation of chondrocytes (e.g., mesethmoid) in HS is observed as high-density area in PCX-CT. CS, Carnegie stage; HS, histological section; PCX-CT, phase-contrast CT; AT, ala temporalis; AP, alar process; B, basisphenoid; ME, mesethmoid; R, root of orbitosphenoid; TN, trigeminal nerve.

Specimens between Carnegie stage (CS) 17 and 23 (Table 1 and Supplementary Table 1) were observed using HS and PCX-CT. The sphenoid in embryos is comprised of 12 primary cartilaginous elements: the orbitosphenoid (2), presphenoid (2), mesethmoid (1), basisphenoid (1), alar process (2), ala temporalis (2), and medial pterygoid (2) (the number within parentheses represents the number of each element) (Schema in Supplementary Fig. 1)^26^. First, we clarified the timing of the first appearance of each element. We simply counted the mesenchymal cell condensation of each element, including slight cellular condensation which indicates that the element had just appeared. If it was positive, it was counted as 1, and the numbers of each stage were added up to determine the sum (Table 2 and Supplementary Fig. 2). This observation was performed in the same way using both HS and PCX-CT. The results showed that the first element was the orbitosphenoid, and the last was the medial pterygoid. All elements except for the medial pterygoid appeared completely until CS21. There were no obvious differences between the right and left sides for the bilateral elements. As shown in Table 2, there were a few discrepancies between the timing of appearance in HS and PCX-CT; most elements had a slightly higher rate of appearance in HS than in PCX-CT at all stages. The medial pterygoid was the only element that had a higher appearance rate in PCX-CT than in HS: in CS21, it was observed in 2 out of 10 embryos with HS and 3 out of 7 embryos with PCX-CT. Additionally, in CS22, it was observed in 3 out of 10 embryos with HS and 3 out of 6 embryos with PCX-CT. Figure 1 shows the basisphenoid region as an example of how the sphenoid in CS20 was observed using HS and PCX-CT. In the HS figure, the basisphenoid consisted of a chondrified matrix surrounded by the perichondrium (Fig. 1a, arrowhead), which was observed in PCX-CT as a low-density area surrounded by a high-density outline (Fig. 1b, arrowhead). In contrast, chondrocyte proliferation areas in HS were exhibited as high-density areas in PCX-CT. For example, the mesethmoid was observed as a mesenchymal cell condensation without perichondrium in HS, and as a high-density area with the same shape in PCX-CT. Thus, all elements observed in HS were also observed in PCX-CT at every stage (Supplementary Fig. 3, several key elements were labelled). However, some elements in PCX-CT required a histological guide in advance to be detected correctly (e.g., the ala temporalis in Supplementary Fig. 4).

**Table 1:** Sample numbers (see Supplementary Table 1 for ID and crown-rump length).

CS	HS	PCX-CT
17	12	7
18	10	8
19	10	12
20	10	10
21	10	7
22	9	6
23	4	7

*CS* Carnegie stage, *HS* histological section, *PCX-CT* phase-contrast CT.

**Table 2:** Appearance rate of the sphenoid elements.

CS	Lateral cartilage								Prechordal cartilage		Hypophyseal cartilage
	Orbitosphenoid		Ali sphenoid				Medial pterygoid process		Presphenoid	Mesethmoid	Basisphenoid
			Alar process		Ala temporalis						
	Left	Right	Left	Right	Left	Right	Left	Right			
**Histological section**											
17	10/12	9/12	0	0	0	0	0	0	0	0	7/12
18	10/10	10/10	2/10	2/10	7/10	7/10	0	0	8/10	0	10/10
19	10/10	10/10	9/10	9/10	6/10	6/10	0	0	10/10	7/10	10/10
20	10/10	10/10	10/10	10/10	10/10	10/10	0	0	10/10	9/10	10/10
21	10/10	10/10	10/10	10/10	10/10	10/10	2/10	2/10	10/10	10/10	10/10
22	9/9	9/9	9/9	9/9	9/9	9/9	3/9	3/9	9/9	9/9	9/9
23	4/4	4/4	4/4	4/4	4/4	4/4	3/3*	3/3*	4/4	4/4	4/4
**Phase-contrast CT**											
17	2/7	2/7	0	0	0	0	0	0	0	0	0
18	6/8	5/8	1/8	1/8	0	0	0	0	1/8	0	2/8
19	10/12	9/12	9/12	9/12	6/12	6/12	0	0	10/12	8/12	11/12
20	10/10	10/10	10/10	10/10	10/10	10/10	0	0	10/10	9/10	10/10
21	7/7	7/7	7/7	7/7	7/7	7/7	3/7	1/7	7/7	7/7	7/7
22	6/6	6/6	6/6	6/6	6/6	6/6	3/6	2/6	6/6	6/6	6/6
23	7/7	7/7	7/7	7/7	7/7	7/7	7/7	7/7	7/7	7/7	7/7

*In one specimen of CS 23 (#2530), several slices around the medial pterygoid process were missing.

**Figure 2:**
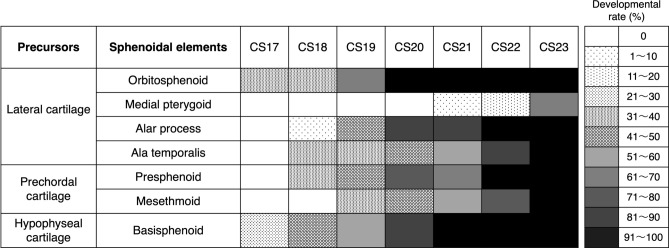
Precise development of each element of the sphenoid. In histological sections, elements that were still cellular condensation were counted as 0.5, and those that produced cartilaginous matrix were counted as 1. Then, the value in each stage was summed up and divided by the number of all specimens, and the developmental rate of each element was calculated. A specific color or pattern was assigned to each 10% of the developmental rate.

Second, we examined the precise development of each element using HS. We counted the elements with cellular condensation (regarded as “under development”) as 0.5, and the elements with chondrified matrices surrounded by the perichondrium (regarded as “developed”) as 1. Then, we summed up the value for each stage, divided the sum by the number of all specimens, and calculated the developmental rate for each element of each stage. In addition, we created a chart by assigning a specific color or pattern to each 10% of developmental rate. As for bilateral elements, the values of both the right and left sides were combined. The results are shown in Fig. 2. According to the figure, each element required two to four stages from the beginning of appearance to the completion of matrices. When we focused on each precursor, the prechordal cartilage seemed to have a slightly later timing of appearance compared to the other two.

**Figure 3:**
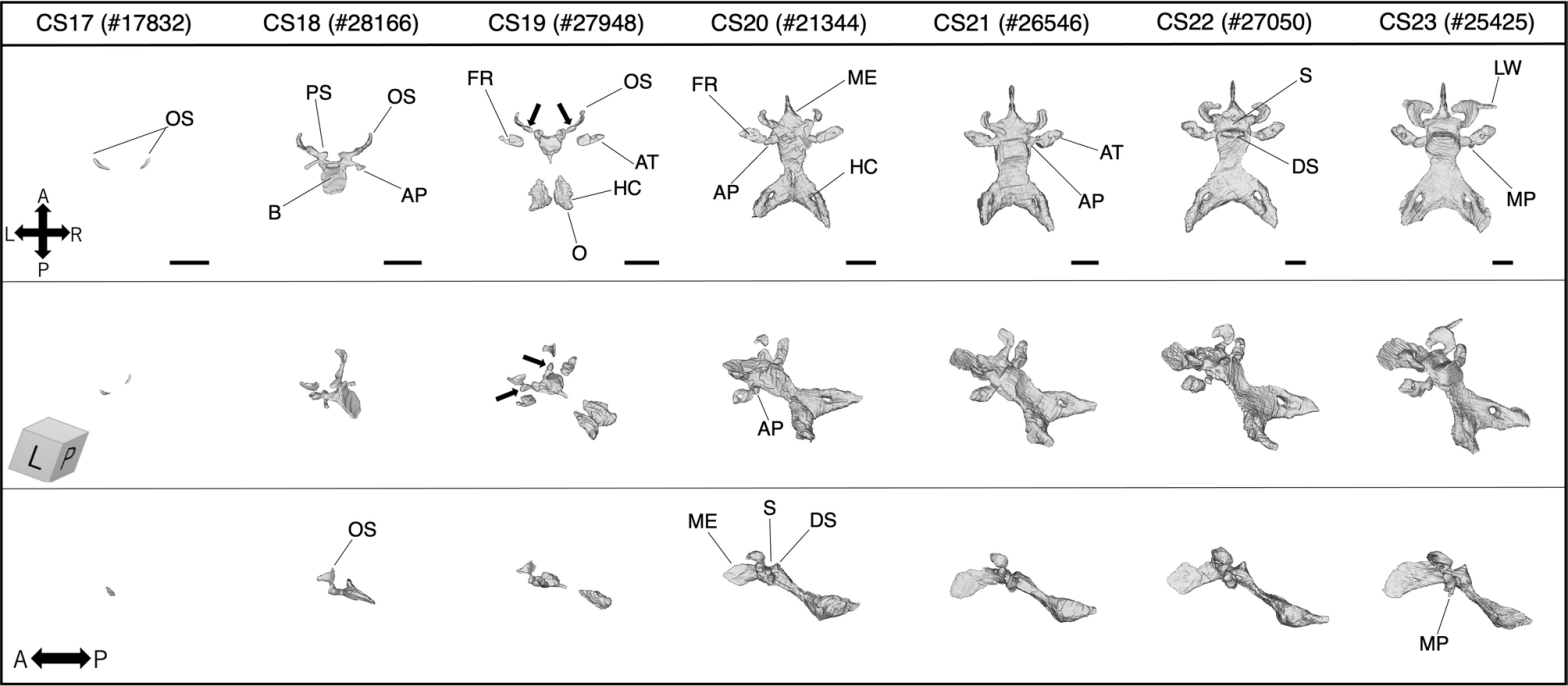
The 3D models reconstructed using PCX-CT. Typical appearance of the sphenoid (including the occipital and the nasal septum) in each stage (black bar: 1 mm). Note that these are not necessarily corresponding to the findings recorded in Table 2 and Fig. 2 which are the results of HS observation. Arrows (CS19) indicate a structure that articulates with the orbitosphenoid and presphenoid, which is called “postorbital root of the orbital wing” or “hypochiasmatic alae” in previous reports. HS, histological section; PCX-CT, phase-contrast CT; AT, ala temporalis; AP, alar process; B, basisphenoid; DS, dorsum sella; FR, foramen rotundum; HC, hypoglossal canal; LW, lateral part of the lesser wing; ME, mesethmoid; MP, medial pterygoid process; O, occipital; OS, orbitosphenoid; PS, presphenoid; S, sella tunica.

The 3D models representing the typical appearance of the sphenoid at each stage are shown in Fig. 3. They include the occipital and nasal septum, which could not be distinguished from the basisphenoid and mesethmoid, respectively. Based on Figs. 2 and 3 and Table 2, we summarized the developmental process of the embryonic sphenoid (Table 3).

**Table 3:** Summary of the developmental process of the sphenoid during the embryonic period.

CS	Summary
17	The orbitosphenoid (OS) appears first, followed by the basisphenoid (BS)
18	OS and BS have stronger cellular condensation The presphenoid (PS) and alar process (AP) develop from the anterior and the back lateral of BS, respectively Mesenchymal cells around the maxillary nerve begin to proliferate (ala temporalis [AT] condensation)
19	An unclear condensation of the mesethmoid (ME) appears in front of PS The foramen rotundum (FR) begins to develop as a bottom half-completed hole within AT Cartilaginous matrix and perichondrium are little formed at this stage Almost all elements still do not have connections with each other
20	OS bends and begins to wrap around the optic nerve to form the optic canal PS fuses in the middle and continues to ME OS and BS have cartilaginous matrix and perichondrium Most elements connect with each other
21	In a few specimens, the medial pterygoid process (MP) develops from the inferomedial of AT FR completes its entire hole ME extends longer toward anterior by connecting with the nasal septum All elements fuse each other, and the boundaries between them become invisible
22, 23	OS enlarges and bends along the optic nerve, and the optic canal is almost fully encircled The lateral part of the lesser wing appears as an unclear and sharp condensation outward from OS in some specimens MP extends longer toward the oral cavity Most elements have massive matrices and clear perichondrium

In CS17, a pair of small mesenchymal cell condensations first appeared in the space between the eye primordium and the third ventricle, which later becomes the orbitosphenoid. In a few specimens, the basisphenoid, which was still a thin and unclear cell condensation, was observed in the center of the rostral side of the hypophyseal infundibular.

In CS18, the orbitosphenoid and basisphenoid were denser and could be easily distinguished. The presphenoid, alar process, and ala temporalis individually appeared as unclear condensations. The presphenoid appeared as bilateral processes on the anterior lateral basisphenoid. The alar process appeared backward from the lateral site of the basisphenoid. Mesenchymal cells around the maxillary branch of the trigeminal nerve began to proliferate (ala temporalis cell condensation).

In CS19, the outline of each condensation was clear. The anterior part of the presphenoid was separated into two bilateral parts. In front of the presphenoid, an indistinct condensation that would later become the mesethmoid also appeared, but it had no attachment with the presphenoid yet. The foramen rotundum began to be observed within the ala temporalis, of which only the bottom half was formed. All elements of the sphenoid, except for the medial pterygoid, existed in this stage, but as separated structures. The 3D models from CS19 to 20 showed that the sphenoid grows not by extending from one cartilage, but by a combination of enlarging and fusing of each element erupting individually. A nodular structure that articulated the orbitosphenoid and presphenoid was observed in the 3D model (Fig. 3, CS19, arrow), and this appeared to occur individually from its own condensation.

In CS20, most elements were connected with each other. The bilateral parts of the presphenoid fused in the midline and then began to connect with the mesethmoid which had developed as a clear triangular condensation. In some specimens, the nasal septum appeared as a very thin cell condensation on the anterior mesethmoid. The alar process articulated the ala temporalis with the basisphenoid. The foramen rotundum was observed in most specimens. The maxillary nerve pre-existed, and the ala temporalis arose from the caudal site of the nerve from CS19, formed a U-shape, and almost encircled the nerve until CS21. The hypoglossal canal followed the same way to form the surrounding of the hypoglossal nerve. The gradual formation of the neural foramina was well grasped using 3D models, but hardly using HS. Moreover, in this stage, the optic canal began to wrap the optic nerve, and the hypophyseal fossa deeply recessed toward the ventral side by enfolding the infundibular process.

In CS21, the medial pterygoid process began to appear. It arose from the medial site of the temporalis and extended longer and downward to the primitive oral cavity in later stages. Cartilaginous matrix production and perichondrium formation occurred first in the orbitosphenoid, followed by the basisphenoid.

In CS22 and 23, most of the elements had almost or completely formed cartilaginous matrix and held a distinct and thin perichondrium. The orbitosphenoid further enlarged anteriorly and inferiorly along the optic nerve, and the optic canal was almost fully encircled. The nasal septum and ethmoid developed anteriorly and downwards. In some specimens, an unclear and sharp outward condensation appeared from the lateral projection of the orbitosphenoid, which would become the lateral part of the lesser wing (intermembranous bone).

The 3D models also showed the following differences in growth among the elements. Cartilage formation was almost complete at CS20, but the orbitosphenoid and mesethmoid (elements anterior to the sella) continued to grow in upward and anterolateral directions, and anterior and inferior directions, respectively, with dynamic shape change even after CS20. On the other hand, the basisphenoid, alisphenoid, and occipital (elements posterior to the sella) grew only in size while retaining their shape after CS20. That is, the shape of the anterior elements was determined later than that of the posterior elements.

**Figure 4:**
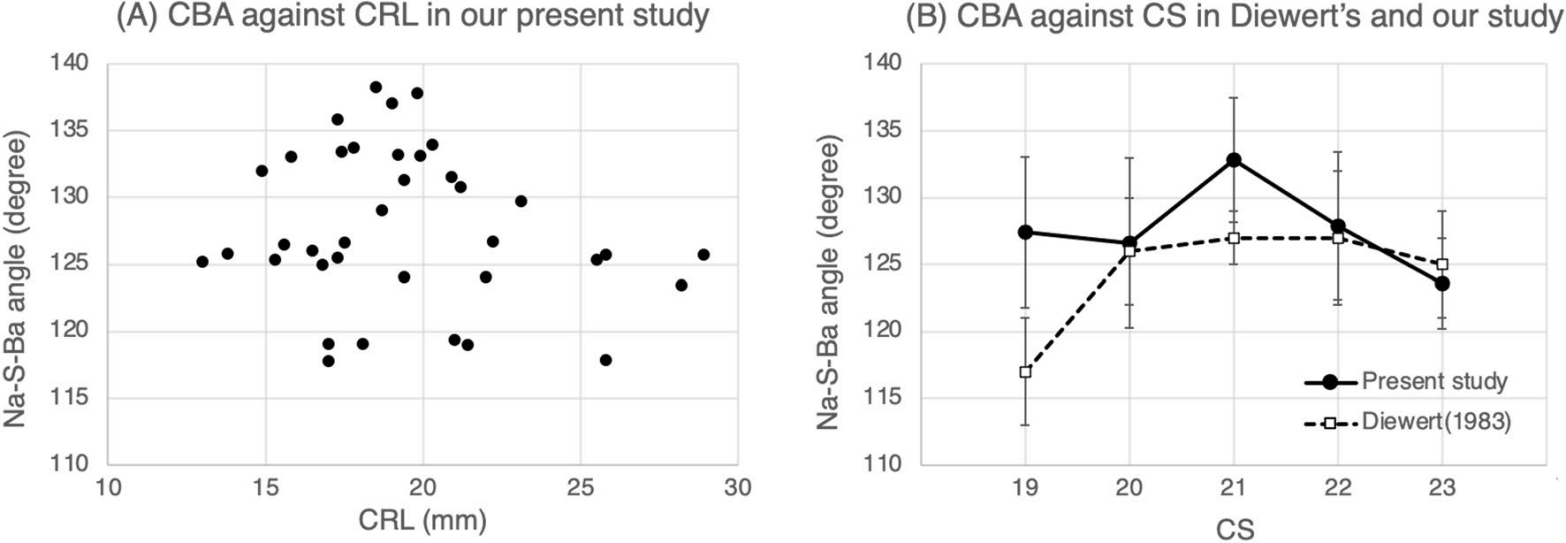
(**A**) Scatter plot of CBA against CRL. The distribution of each sample data has a high degree of variability. An approximate curve or line cannot be applied because of a low coefficient of determination for this scatter plot. (**B**) The mean and SD of CBA in both Diewert’s and our study are shown in the graph based on Table 4A. Our result shows an acute increment of CBA at CS21, whereas Diewert’s result shows flattening after CS20. Comparing only our present results in the two graphs, an acute increment of CBA at CS21 (**B**) fades away when it is against CRL (**A**). CBA, cranial base angle; CRL, crown-rump length; CS, Carnegie stage; SD, standard deviation.

Table 4 and Fig. 4 show the results of the cranial base measurements in sagittal sections during the late embryonic period. The cranial base angle (CBA) was recorded as the angle between the nasion (Na), sella (S), and basion (Ba), and the length of the anterior and posterior cranial base as the distance between Na and S (Na-S), and S and Ba (S-Ba), respectively. The intraclass correlation (ICC) values calculated for the Na-S distance, S-Ba distance, and Na-S-Ba angle were 0.99, 0.96, and 0.87, respectively, suggesting high measurement reliability. Table 4A shows the mean and standard deviation (SD) of the cranial base lengths and CBA of each stage, including 37 specimens from the present study and 37 specimens cited from Diewert’s histological study^37^. Table 4B shows the significant difference between stages calculated using analysis of variance (ANOVA). Significant differences were observed in Na-S and S-Ba distances, but not in the Na-S-Ba angle either in Diewert’s or our study (*P* = 0.317 in our study). Figure 4A shows a scatterplot of CBA against CRL for our specimens. The data distribution of each sample had a high degree of variability, and an approximate line could not be applied because of the low coefficient of determination for this scatter plot (R^2^ = 0.0293). Figure 4B shows the mean and SD of CBA at each stage based on Diewert’s and our value which are recorded in Table 4A. In this graph, the CBA of our study showed a steep increment with the largest mean angle of 132.8° at CS21, which reduced toward CS22 to 23. In contrast, in Diewert’s study, it was the smallest at 117° at CS19 and remained constant at approximately 127° in later stages. Comparing our results in Fig. 4A and B, we observed that an acute increment of CBA at CS21 (B) faded away when it was against CRL (A).

**Table 4:** Measurements of the cranial base in sagittal sections of human embryos during the late embryonic period.

CS	19		20		21		22		23	
Number of samples	Present study (n = 11)	Diewert (n = 10)	Present study (n = 9)	Diewert (n = 9)	Present study (n = 7)	Diewert (n = 7)	Present study (n = 5)	Diewert (n = 7)	Present study (n = 5)	Diewert (n = 4)
**(A)**										
Mean CRL (mm)	16.34	18.1	17.81	21.2	20.00	22.8	21.54	25.5	26.45	29.3
Na-S Distance (mm)	2.44 ± 0.25	2.3 ± 0.4	2.71 ± 0.11	2.6 ± 0.3	2.93 ± 0.19	3.4 ± 0.4	3.27 ± 0.92	3.6 ± 0.3	4.04 ± 0.60	4.5 ± 0.6
S-Ba Distance (mm)	2.33 ± 0.18	2.5 ± 0.4	2.65 ± 0.06	2.6 ± 0.5	2.82 ± 0.22	3.2 ± 0.3	3.05 ± 0.26	3.3 ± 0.3	3.60 ± 0.31	3.6 ± 0.3
Na-S-Ba Angle (°)	127.4 ± 5.62	117 ± 4	126.6 ± 6.34	126 ± 4	132.8 ± 4.67	127 ± 2	127.9 ± 5.51	127 ± 5	123.6 ± 3.36	125 ± 4

				Present study				Diewert’s study		
**(B)**										
Na-S Distance (mm)				*P* < 0.05				*P* < 0.001		
S-Ba Distance (mm)				*P* < 0.05				*P* < 0.001		
Na-S-Ba Angle (°)				NS (* P* = 0.317)				NS		

(A) Mean ± standard deviation (SD) for Diewert’s and our studies are presented in this table. Each value of Diewert’s study was obtained by dividing the original value by 36 (see *Materials and methods*). (B) Significant differences between stages were calculated using the F-test of the analysis of variance. A *P*-value greater than 0.05 was defined as nonsignificant (NS).

*CS* Carnegie stage, *CRL* crown-rump length, *Na* nasion, *S* sella, *Ba* basion.

## Discussion

In the present study, we observed the initial morphogenesis of the human sphenoid in detail using HS and PCX-CT. Additionally, we visualized the 3D models of continuous shape changes of the embryonic sphenoid. The 3D models demonstrated several new findings, including the process of some neural foramina or the differences between the shape change in the anterior and the posterior sphenoid. Moreover, we measured CBA in an accurate sagittal section using PCX-CT, and we revealed that CBA against CS had a peak at CS21 whereas CBA against CRL showed no striking peak. There are several methodological limitations to the present study. First, we could not investigate HS with sagittal and horizontal slides due to a lack of samples, and there might be some unobserved structures. Second, the observations in this study were limited to a narrow period of human development and are focused on the sphenoid, not all regions in the chondrocranium.

According to this study, the differences between HS and PCX-CT in observing the embryonic sphenoid can be described as follows. HS is suited for observation of local microanatomy with its high resolution, which allows observation of the properties of individual cells, such as the size, shape, cell matrix, or nucleus. On the other hand, possible disadvantages are that it does not enable observation of intricate structures because one sample is simply sliced in a single direction (e.g., horizontally, sagittally, or coronally), and that samples are sometimes sliced in the diagonal direction. PCX-CT does not have sufficient resolution to discriminate at the cellular level and to immediately recognize the primitive and unclear structures. Instead, PCX-CT is suitable for simultaneous observation from multiple directions, which can compensate for the inferiority of the lower resolution compared with that of HS, and to visualize any objects with complicated structures, such as the protuberances, recessions, or holes. Consequently, in this study, a combination of these two methods allowed us to obtain more information than we could have obtained with one.

The order of appearance of mesenchymal condensations observed in our study (Table 2 and Fig. 2) was consistent with previous studies by Fawcett^28^ and Tanaka^38^, that is, the orbitosphenoid is the first element to arise, followed by the posterior elements. In addition to previous studies, our study was able to confirm the entire order of development of all elements using two observation methods. As 3D models (Fig. 3) suggest, the primary elements do not arise at the same position as ossification centers in older fetuses. For example, it has been reported that the postsphenoid has four ossification centers^26^, whereas only a single condensation was observed in the embryonic period.

Furthermore, 3D models visualized the process of forming several neural foramina in the chondrocranium. The foramen rotundum and hypoglossal canal developed in the same way as the mental foramen^39^, that is, the nerves appeared first, followed by mesenchymal cells surrounding the nerves from the U-shaped to the entire circle gradually. This indicates that the morphogenesis of the neural foramen is common among various regions. It has been speculated that bone formation can be influenced by signaling molecules present in adjacent tissues; however, the detailed mechanism by which the bony structure develops to surround the nerve remains unclear^40^.

Comparing our 3D models with those of the chondrocranium in mice^41^, the following differences were found. In the human sphenoid, the orbitosphenoid is noticeably developed in size and thickness, larger than the ala temporalis at the end of the embryonic period, but in mice, it is smaller than the ala temporalis. The foramen rotundum and optic canal are oriented toward the anterior in humans, and lateral in mice. Moreover, the interval length between the orbitosphenoid and ala temporalis in humans is evidently shorter than that in mice. The sphenoid of human is more uneven (e.g., the dorsum sella is prominent), whereas that of mice appears to be flat. Note that these results do not necessarily reveal morphological differences between the two species correctly, because there are differences in imaging equipment and the use of contrast agents. Our study revealed that human and mice sphenoid possess common elements but clearly differ in morphology, although it was not possible to perform quantitative comparative analysis between the sphenoid of humans and mice.

In our 3D models, we observed that a pair of cartilages articulated with the orbitosphenoid and presphenoid (Fig. 3, CS19, arrows) and subsequently became the posterior root of the lesser wing. De Beer^24^ has mentioned that this cartilage has various locations among different mammals, and McBratney-Owen et al.^42^ emphasized that it may orientate the attachment of the lesser wing to the presphenoid. In mice embryos, it is named the “hypochiasmatic cartilage.”^41,42^ However, as for human embryos, it has inconsistent names, such as “hypochiasmatic alae”^29^ or “postorbital root of orbital wings,”^30^ and has not been included in the primary elements of the sphenoid (shown as broken circles in Supplementary Fig. 1). As our study indicates, this cartilage arises independently during the embryonic period as well as other elements. Therefore, from an embryological point of view, we suppose that it should be given a standardized name, such as “hypochiasmatic process” and be included in the primary elements. Indeed, it would also be necessary to consider its evolutionary history and homology with other animals to certify this name formally, similar to other elements^43^.

Our results demonstrated that there was a slight but noteworthy difference in the angulation tendencies between CS and CRL. A steep increment in CBA at CS21 when CS was used as an explanatory variable (Fig. 4B) slacked off in the graph against CRL (Fig. 4A). Here, it should be noted that CRL is just a simple value indicating body length enlargement, whereas CS is a classification according to the developmental level reflecting various embryological events that occur in the body. Consequently, what is implied here is that the temporary flattening of CBA at CS21 might be related to any of the embryological events which occur around craniofacial regions. One of the possible events that can affect angulation is forebrain enlargement. It has been reported that the forebrain grows rapidly during CS21 to 22 in 2D area^44^ and 3D volume^45^. The elevation of the palatine process, which occurs from CS21 to 23^37^, may be considered as another possible event that may indirectly affect CBA by alterations in the amniotic fluid pressure, flow rate, and oral pressure^46^. As for notable events in the face during these stages, a convergence of the eyes takes place dynamically^47^. Thus, in humans, as a series of significant embryological events occur around CS21, we assume that the distinctive change of CBA against CS had a biological significance rather than it appeared accidentally. Our study on CBA includes several limitations as follow. One limitation is the differences in the condition of specimen fixation. This can affect the thickness of the skin and soft tissue, which can produce errors in the measurement of CRL or the cranial base length. The other is the unintentional contamination with unknown morphological anomalies.

The development of the sphenoid is interesting, either ontogenically or evolutionally. Ontogenically, two different origins divided by the sella become the anterior and posterior sphenoid^42,48,49^ and are known to have different growth speeds^50,51^. From an evolutionary point of view, the posterior cranial base is considered a highly preserved structures from very early mammals^43^, while the anterior is considered an evolutionarily new and potent region for inducing human-specific facial growth^25,52^. In this study, we found a small but interesting difference between the morphogenesis in the anterior and posterior sphenoid, that is, shape changes lasted longer in the anterior than in the posterior sphenoid (Fig. 3, from CS20 to 23). This may indicate that the anterior sphenoid has more plasticity and undergoes remodeling. Consequently, this seems to support the hypothesis that the anterior part has a potential to create specific features of the human face by such plasticity and instability during evolution. Furthermore, in patients with congenital craniofacial anomalies, it has been suggested that deformities in the cranial base are likely to be more prevalent in the anterior, including the sphenoid and ethmoid bones, than the posterior. The greater plasticity of the anterior sphenoid than the posterior, as indicated by our findings, may also be suggestive of a susceptibility of the anterior sphenoid to craniofacial anomalies. Our results possibly support this hypothesis but are not sufficient to certify it. Further 3D quantitative multivariate analysis of the morphology of not only the sphenoid but also the whole chondrocranium may allow us to elucidate the pathogenesis of craniofacial anomalies. It would be important to investigate inter-individual differences among the same-staged specimens to discuss this potential of the anterior sphenoid. However, according to our observation, it was difficult to distinguish whether the morphological differences among the same-staged specimens were caused by a developmental difference or by an inter-individual difference. Further study using quantitative morphological analysis, such as geometric morphometrics, may enable us to detect inter-individual subtle features, which provides insights into what determines human facial morphology during development or evolution.

To discuss the morphogenesis, one must also consider the theory called as the “muscle–tendon-bone complex^53^” which has been reported recently. According to this theory, differences in the function and load of the muscle attached to the bone could drive the morphogenesis and maintenance of the bone itself. For example, Yamamoto et al.^54^ revealed through the investigation of mice embryos that the medial pterygoid process (bone) rapidly grew up after the tensor veli palatini (muscle) combined to the palatine aponeurosis (tendon) and suggested that the bone growth occurs through the interaction of bone, muscle, and tendon^54,55^. Further investigation of the interaction between bone and muscle including molecular biologic studies would be important to elucidate the morphogenesis of the sphenoid^55^ as well as the human skull.

To the best of our knowledge, this study is the first to visualize continuous 3D morphogenesis of human embryonic sphenoid which is located in the center of the human chondrocranium. The chondrocranium is supposed to be a primary mold in planning the cranial morphology, which play a role as a temporary scaffold for later membranous bone growth to support the brain and other sensory organs^51,56^. As the human skull has a highly complicated shape which holds various structures, organs, and muscles, the elucidation of the human skull morphogenesis requires a multi-viewpoint approach. We believe that the integration of recent advances in 3D imaging techniques and conventional histological studies will enable the advanced investigation, including molecular or genetic biological study, which will help us understanding the human skull morphogenesis.

## Materials and methods

### Specimens

All specimens belonged to the Kyoto Collection owned by the Congenital Anomaly Research Center of Kyoto University, which is one of the largest collections of human embryos and fetuses. It includes more than 45,000 embryos and fetuses, containing specimens from both normal and abnormal embryos. These have been obtained with induced abortions by designated gynecologists under the Maternal Protection Act of Japan. Specimens without any distinct anomalies in their appearances were chosen between CS17 and 23 (Table 1 and Supplementary Table 1). For HS, 65 embryos were selected, with CRL ranging from 9.9 mm to 27.3 mm. For the PCX-CT study, 57 formalin-fixed embryos were selected, and CRL ranged from 8.5 mm to 28.9 mm. There were several specimens without CRL measurement because it could not be measured correctly due to body damages, and they were recorded as “not available (NA).” The age of human embryos is often represented as gestational age (GA), which is usually based on the mothers’ self-reported menstrual date. According to the menstrual date, GA of our samples was 28 to 185 days, whereas our samples used in the present study were staged from CS17 to 23 according to the features of their external appearances and corresponded to approximately 48 to 56 days (6 to 8 weeks). Hence, the age estimation from the menstrual date could be unreliable for our samples. Therefore, in our study, CRL instead of GA was used as the growth parameter of embryo samples.

### Observation of histological sections

The specimens were sliced into a horizontal section of 10 μm thickness, stained with hematoxylin and eosin, and then, observed using an Olympus BX51 microscope (Olympus, Tokyo, Japan) at low and high magnifications.

### Imaging device and acquisition of image data

The PCX-CT system was set at the beamline BL-14C2 (the Photon Factory (synchrotron facility), KEK, Tsukuba, Japan) and used for imaging the samples using monochromatic synchrotron radiation (SR). The system has a skew-symmetric two-crystal X-ray interferometer (STXI) to detect the phase-shift that occurs when the X-ray passes through a sample^57^. The specifications of this imaging study were 17.8 keV for SR, 60 mm × 30 mm for field view, and 13 µm for pixel size. The phase-contrast tomogram was obtained with three-step image processing (see details in Yoneyama et al*.*^36^) and transformed into Digital Imaging and Communications in Medicine (DICOM) format.

### Observation and 3D reconstruction using phase-contrast CT

DICOM data were transferred into the 3D Slicer^58^ and Horos™ (Horos Project, Annapolis, MD, USA) software programs, and the samples were observed. To visualize the embryonic sphenoid during development, 3D models of the sphenoid were reconstructed from PCX-CT data using the “segmentation” tool incorporated within 3D slicer. We manually segmented the sphenoid in each slice and then stacked the segments to form a 3D model (Supplementary Fig. 4). Finally, we minimally smoothed out their surfaces using a smoothing tool to make it easy to grasp the morphology of each element.

### Measurement of the cranial base angle and length

CBA and the length of the anterior and posterior cranial base were measured using the Horos™ software. We used 37 samples, which were selected from Table 1 and specimens with any cutaneous damages in the facial region were excluded. The definition of each measurement point was set in the midsagittal section, based on Diewert’s study in 1983^37^. Supplementary Fig. 5 shows the measured points. Cutaneous nasion (Na) was defined as the most recessed point on the skin above the nose with the largest curvature. The sella (S) was located at the center of the hypophysis. The basion (Ba) was defined as the posterior end of the basioccipital region. CBA was recorded as the angle of Na-S-Ba and the length of the anterior and posterior cranial base as the distance between Na-S and S-Ba, respectively. The measurement for each value was examined twice by one examiner (N.U.), at least 48 h apart from the first examination. ICC was calculated as ICC1 (intra-rater reliability) using R (package “psych”). The mean and SD were calculated for each measurement at each stage. Each value was compared with Diewert’s previous results. Regarding the results of the cranial base length of the previous study, each value was obtained by dividing the original value by 36 (each result of the previous study had been measured after magnification 36 times for the actual embryo size. see Diewert^37^). The F-test of ANOVA was performed for the Na-S, S-Ba distances, and Na-S-Ba angle to analyze significant differences among stages and was recorded as significant when the p-value was less than 0.05.

### Ethical considerations

This study was approved by the Ethics Committee of the Graduate School of Medicine and Faculty of Medicine, Kyoto University (approval no. R0316 and R0347), and was performed in accordance with the Ethical Guidelines for Medical and Biological Research Involving Human Subjects. The identification of samples used in this study has been anonymized, and personal information cannot be identified by any researchers. Verbal informed consent with written statement was obtained from the parents for all samples when donating them in the 1960–1990s.

## Supplementary Information

Supplementary Information.

## Data Availability

The datasets analyzed during the present study are available from the corresponding author on request.
