# Author Correction: The first 3D analysis of the sphenoid morphogenesis during the human embryonic period

**DOI:** 10.1038/s41598-023-33506-3

**Published:** 2023-04-24

**Authors:** Natsuko Utsunomiya, Motoki Katsube, Yutaka Yamaguchi, Akio Yoneyama, Naoki Morimoto, Shigehito Yamada

**Affiliations:** 1 Department of Plastic and Reconstructive Surgery, Graduate School of Medicine, Kyoto University, 54 Kawahara‑cho, Shogoin, Sakyo‑ku, Kyoto, 606‑8507 Japan; 2 Congenital Anomaly Research Center, Graduate School of Medicine, Kyoto University, Kyoto, Japan; 3 SAGA Light Source, Saga, Japan; 4 Human Health Sciences, Graduate School of Medicine, Kyoto University, Kyoto, Japan

Correction to: *Scientific Reports* 10.1038/s41598-022-08972-w, published online 28 March 2022

The original version of this Article contained errors in the Abstract, the Materials and methods section, and the Supplementary Information.

In the Abstract,

“We examined 54 specimens using HS and 57 specimens using PCX-CT, and summarized the initial morphogenesis of the sphenoid during Carnegie stage (CS) 17 to 23.”

now reads:

“We examined 65 specimens using HS and 57 specimens using PCX-CT, and summarized the initial morphogenesis of the sphenoid during Carnegie stage (CS) 17 to 23.”

In the Materials and methods section, under the subheading ‘Specimens’,

“For HS, 54 embryos were selected, with CRL ranging from 9.9 mm to 27.3 mm.”

now reads:

“For HS, 65 embryos were selected, with CRL ranging from 9.9 mm to 27.3 mm.”

Finally, in Supplementary Figure 1 legend, the abbreviation of “mesethmoid” was given incorrectly.

“MS”

now reads:

"ME".

The original Article and accompanying Supplementary Information file have been corrected.

