# Supplementary material for: The first 3D analysis of the sphenoid morphogenesis during the human embryonic period
Source: Sci Rep. 2022 Mar 28;12:5259. doi: 10.1038/s41598-022-08972-w (PMC8960892; doi:10.1038/s41598-022-08972-w)

Supplementary Table 1

Sample ID and CRL (mm) for the present study.

CS, Carnegie stage; CRL, crown-rump length; HS, histological section; PCX-CT, phase-contrast CT; NA, not available value

CS	ID (HS)	CRL (HS)	ID (PCX-CT)	CRL (PCX-CT)
17	111	12	14676	9.5
	505	14.4	14771	8.5
	640	10.5	15022	9.1
	830	11	17805	10.2
	887	NA	17832	9.8
	1440	10.4	19370	9.5
	1583	11.8	20042	10.2
	3239	10		
	3477	11.1		
	3837	9.9		
	4242	14.5		
	33763	12.3		
18	202	15.6	22103	11.9
	1250	13.9	24594	11.6
	1633	13.2	24638	11.6
	2643	12.8	24654	12.7
	2706	14.1	28166	15.9
	3006	15	31870	11.8
	15799	15	32109	10.9
	24992	12	32404	11.5
	25391	13		
	28129	11.8		
19	32	15	16219	17
	829	16	17944	17.8
	2114	17.5	21011	15.3
	2425	17.2	22621	14.9
	3002	18.4	24092	17.3
	4081	15.6	25519	13
	4424	15.2	27948	13.8
	7711	16.5	28447	15.8

	7778	16.4	29160	17.3
	16559	16.3	31686	16.5
			31851	21
			33249	14.8
20	1125	18	19836	17.4
	1770	18.9	20268	18.7
	1950	20	21344	19.4
	2006	15.9	21594	17.5
	4330	18.6	22171	17
	7271	16.9	22403	16.8
	10940	17.7	24649	18.1
	12113	17.9	25322	15.6
	13800	16.5	26354	19.8
	15050	17	32108	17.4
21	291	20	24325	19
	956	20.2	24488	19.9
	1071	20.9	24728	20.9
	1074	20	26546	20.3
	1634	17.4	29762	19.4
	4168	22.3	30652	18.5
	5177	NA	33434	22
	5767	18.3		
	11235	22		
	11315	19.9		
22	776	26	16686	19.2
	2027	20.5	23275	19.8
	2705	20.2	27050	22.2
	3098	20.7	27545	21.2
	5862	21	32163	23.1
	8002	20.8	38466	21.4
	9305	22		
	12246	21.2		
	14814	23.7		
23	2530	24.7	19312	23.1
	4089	24.5	24582	28.9
	4434	NA	25425	25.8

10273	27.3	29137	28.2
		30363	25.8
		52578	25.5
		52817	24.5

Supplementary Figure 1

Schema of primary cartilaginous elements of the sphenoid during human embryonic period.

AT, ala temporalis; AP, alar process; B, basisphenoid; ME, mesethmoid; MP, medial pterygoid process; OS, orbitosphenoid; PS, presphenoid

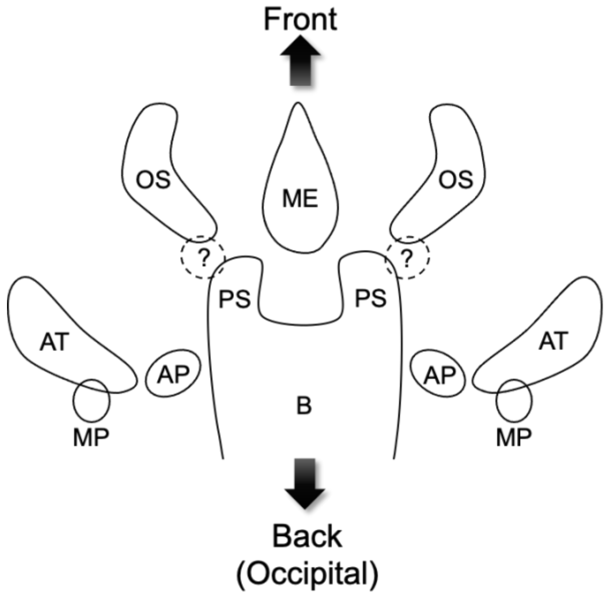

Supplementary Figure 2

Examples of "mesenchymal cell condensation" which was counted as "1" in HS and PCX-CT (for **Table 2** examination).

(A) Cellular condensation of the orbitosphenoid in CS 17 embryo (arrowhead).

(B) Cellular condensation of the ethmoid in CS 20 embryos which has just appeared (above, arrowhead) and has grown afterward (below, arrowhead). We counted both as "1".

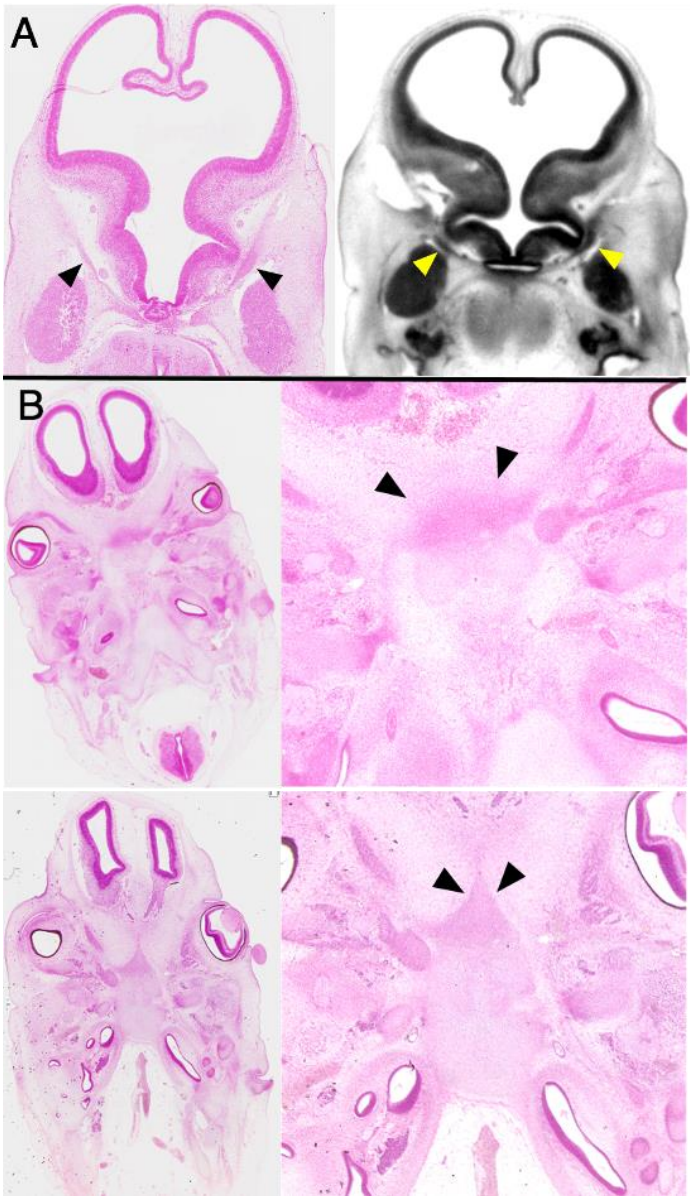

Supplementary Figure 3

Examples of how the sphenoid are observed in each stage using HS and PCX-CT. Samples of HS are displayed in the upper row, and those of PCX-CT are in the lower. All figures show a horizontal slice at the level of the basisphenoid. Asterisk (*) shows the eye primordium. AT, ala temporalis; AP, alar process; B, basisphenoid; H, hypophysis; ME, mesethmoid; OS, orbitosphenoid; PS, presphenoid; R, root of the orbitosphenoid; TG, trigeminal ganglion; TV, the third ventricle.

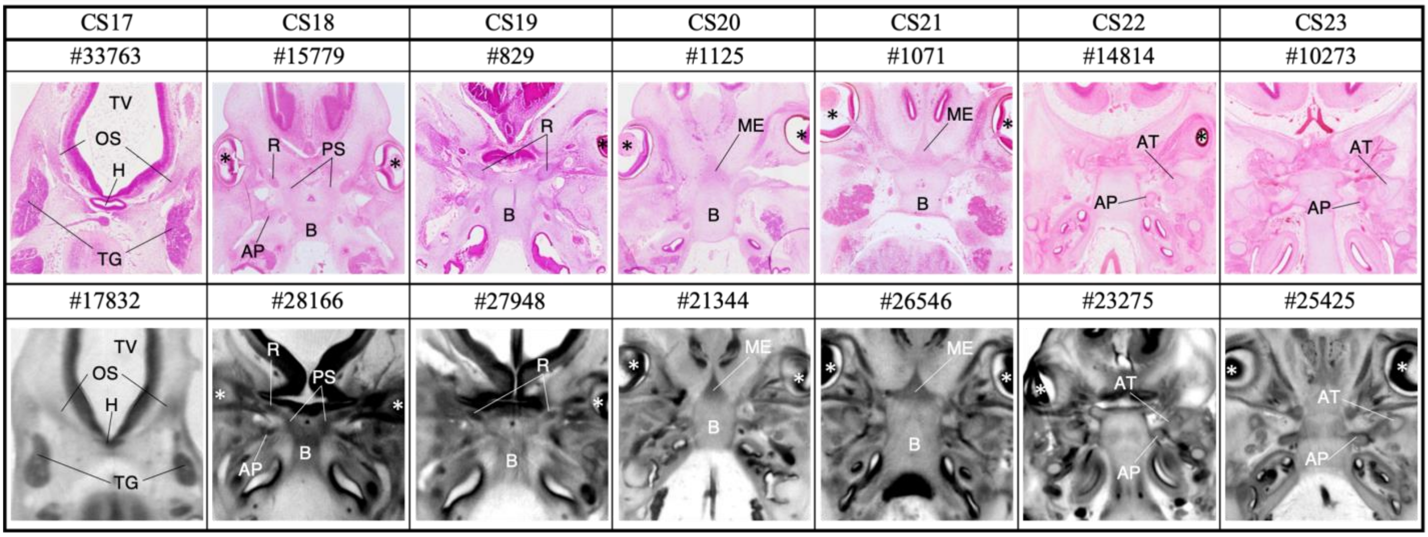

Supplementary Figure 4

Example of manual segmentation of the ala temporalis using 3D slicer (CS21 embryo). Most sphenoid elements were identified only by PCX-CT with no difficulty when we observed them by serial and multidirectional images using DICOM observing tools, while we needed to use HS in advance for identification of some unclear elements as the figure shows. Then, we manually segmentated it with PCX-CT data and stacked to reconstruct 3D models.

AT; ala temporalis, TN; trigeminal nerve.

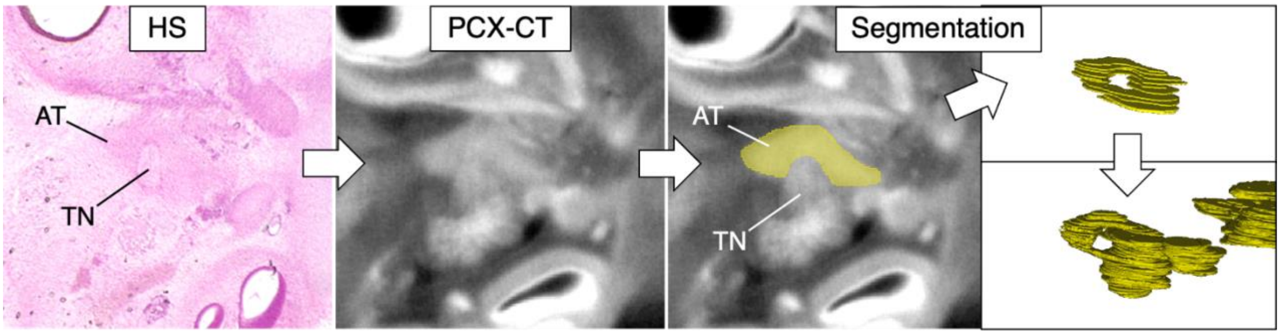

Supplementary Figure 5

Landmarks for cranial base measurement.

This figure shows a sagittal cross section of CS23 embryo (#25425) using PCX-CT and three landmarks for cranial base measurement. Cutaneous nasion (Na): the most recessed point on the skin above the nose with the largest curvature. Sella (S): the center of the hypophysis. Basion (Ba): the most posterior point of the occipital.

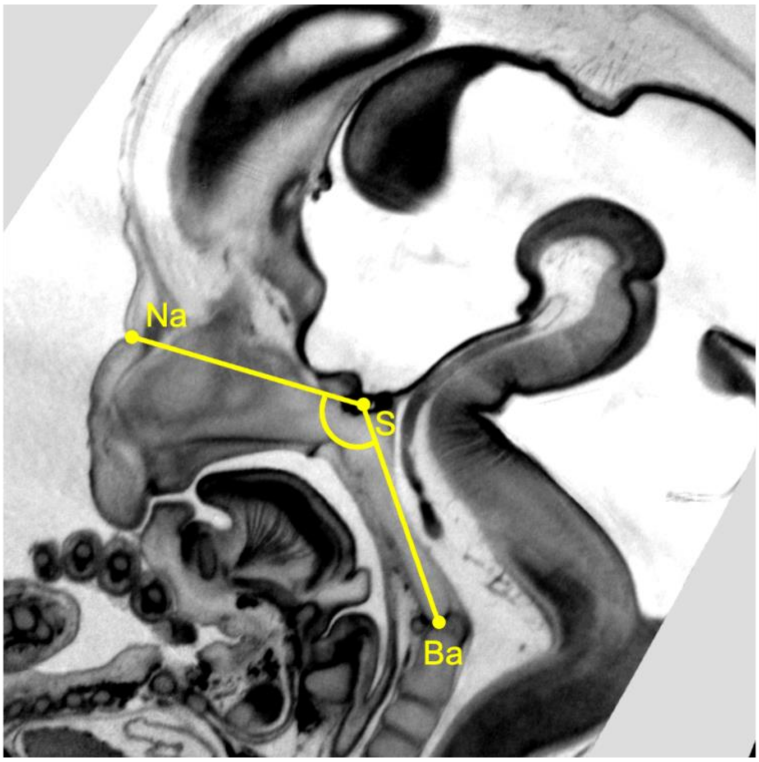

Supplement: Supplementary file 1 — Supplementary Information. [file 41598_2022_8972_MOESM1_ESM.pdf]
